# Building Connection and Resident Understanding of Local Resources Through Community Engagement

**DOI:** 10.5811/westjem.39647

**Published:** 2025-09-01

**Authors:** Hannah Johnshoy, Ashley Pavlic, Sehr Khan, Taylor Sonnenberg

**Affiliations:** * Medical College of Wisconsin, Department of Emergency Medicine, Hub for Collaborative Medicine, Milwaukee, Wisconsin

## Abstract

**Introduction:**

Throughout graduate medical education (GME), it is crucial for learners to not only develop the skills necessary to manage a wide variety of medical conditions, but also to foster personal development and to gain a deeper understanding of the complex and multifaceted needs of our patients. We often refer patients to community sites to address needs such as homelessness, hunger, and domestic violence; however, we frequently make these referrals with only a superficial understanding of what each resource entails.

**Methods:**

To address this issue, our department integrated a two-day Community Engagement Retreat into our curriculum. Twenty-two first-year residents participated in small group visits to three or four community organizations. There, residents engaged with community workers and the public to learn about the services each program offers. This was followed by a session of focused reflection and discussion on how to integrate this new knowledge into our care for patients in the emergency department. At the conclusion of the experience, residents completed an anonymous survey with a response rate of 77.3%.

**Results:**

The results suggest that participants found the sessions highly useful, with 98.6% of residents reporting that they “agreed” or “strongly agreed” that the experiences at the community sites would better allow them to care for patients. They further stated that the program was one of the most impactful elements of their training and highly recommended it to future learners.

**Conclusion:**

This initiative demonstrates the importance and utility of a novel, structured community engagement to begin to address this deficiency in GME and improve patient care.

## INTRODUCTION

Graduate medical education (GME) is a time of significant growth and challenges for emergency medicine (EM) residents. While most programs do an excellent job teaching procedural skills and pathophysiology of disease, we are becoming increasingly aware of the role the social determinants of health (SDoH) play in the varying presentations of our patients.^1^ Importantly, many residents feel this is not well covered in their programs.^2^

The emergency department (ED) is frequently referred to as the “safety net” of the healthcare system.^3^ The SDoH concerns of domestic violence, homelessness, mental illness, and poverty frequently converge to influence our patients’ complex presentations to the ED.^1^,^4^ For example, one study found that 23.5% of respondents said they went to an ED as a “first-stop” site after becoming homeless.^5^ Intervening on SDoH in our patients is vital to providing optimal care,^1^,^6^,^7^ and we commonly find ourselves referring our patients to community services for this support. However, we often make these referrals without fully understanding these services ourselves, limiting our ability to select appropriate resources and to properly counsel our patients. While team members like social workers can be helpful in bridging this gap, they are not always available. Having a general understanding of the resources available in our city to address our patients’ varying SDoH needs is a crucial component of providing complete care, as well as developing our own ability to have professional, compassionate discussions about these issues with them.^7^

In this paper, we describe a focused community engagement retreat for first-year EM residents and evaluate its feasibility and their reception of it. The goal in establishing this retreat was to educate residents on how to address SDoH with their patients, which has been noted as a gap in EM residency training; the retreat was tailored to the community that the EM residents are serving, an important consideration in the design of these types of experiences.^2^,^7^

## METHODS

This retreat was provided for residents within an academic EM program that trains 12 residents per year and serves a major metropolitan area in the Midwest. The retreat was structured to give each resident an opportunity to experience several different community sites. Facilitators reviewed resources commonly used in our area and arranged for small group tours and informational sessions. The retreat was targeted toward first- year EM residents and occurred on the first Wednesday in October of their intern year. The resident class was excused from clinical duties for two days to participate in the retreat and follow-up discussion.

Residents toured three or four organizations known to be used by patients who are seen in our ED, chosen by faculty members who created this experience, mostly leveraging existing relationships. This included groups addressing food insecurity, domestic violence, emergency mental health service, and homelessness (see Table). Residents visited the sites in groups of 4–6. While at the site, residents engaged with community workers and members of the public to learn about the services offered at that location and the population they serve. The day typically included a tour of the facility, informational sessions with managers, and occasionally hands-on participation such as assistance with preparing meals, outreach, or a walk-through of the intake process. Residents spent an average of 2–3 hours at each site.

**Population Health Research Capsule**

What do we already know about this issue?

*While interventions targeting social determinants of health enhance patient care, residency programs lack sufficient training on their practical application*.

What was the research question?

*We examined the feasibility of a community engagement retreat for emergency medicine (EM) residents*.

What was the major finding of the study? *98.6% of respondents reported improved understanding of resources, patient experience, and care capabilities*.

How does this improve population health?

*Community engagement retreats enhance EM residency programs, leading to improved patient outcomes by integrating social determinants of health*.

The site visits were followed by a two-hour session of small-group reflection and discussion between residents and facilitators to process, discuss, and share experiences. In this way, all residents were able to learn about all sites. At the end of the experience, we asked residents to complete an anonymous, voluntary survey that included a Likert scale, which asked residents about their understanding of each community resource and their comfort level in discussing it with patients after touring. The survey also asked for their free-text takeaways from the experience, and their free-text suggestions for improvement. The two faculty members who created the retreat also designed the unpiloted survey (see Figure). So far, two years of survey data have been completed and aggregated to provide overall data. Participating residents were asked about each of the community organizations that they visited. Additionally, qualitative data and quotes were collected from residents in the anonymous surveys to better characterize residents’ views of the retreat. The responses were reviewed and summarized, and representative comments by consensus of all the authors. This project was submitted to our institutional review board (IRB) and determined to be an internal institutional quality improvement project that did not need IRB approval.

## RESULTS

Of 12 residents, 11 participated in the retreat in 2021, and 11 of 12 residents participated in 2023. Seventeen residents (77.3%) responded to the survey. We collected 74 responses about eight community organizations. Feedback from participants was overall extremely positive. Of residents surveyed, 98.6% agreed or strongly agreed that they felt more familiar with resources available, had a deeper understanding of the experiences of the people who use these resources, and felt that they were able to provide better care to patients in the ED after participation in the community engagement retreat (Figure).

The specific quotes provided by residents provided more insight into their experience. In the open response section of the surveys, residents stated that this was one of the most impactful parts of their training and noted that it enhanced their ability to provide better patient care in the ED. One resident said “it was an absolutely fantastic experience—every class in the future should do this.” Another echoed this sentiment:

[It was an] incredible experience. I learned so much about our community and the resources available. Would highly recommend to future classes.

Indeed, the belief by current residents that this retreat should be offered annually was reflected in much of the feedback we received. The overwhelming majority of participants felt that their understanding of the different resources had improved greatly and indicated they felt more confident discussing these resources with patients. Additionally, residents felt that they were better able to not only understand the resources, but also the people who used them. “[I learned the importance of] meeting patients where they are at,” said one resident, after their tour of a homeless outreach center. Another resident noted: “[I] will be more aware of my own implicit biases” after this retreat. One resident noted improved empathy for patients experiencing homelessness.

## DISCUSSION

This is an initial evaluation seeking to address the feasibility of and response to a focused community engagement retreat for first-year EM residents. Initial results suggest that residents did indeed find this retreat to be useful in terms of improving their ability to address the SDoH while on shift in the ED. Discussions revealed that residents felt that their experiences allow for improved rapport with patients and increased confidence in initiating discussions regarding SDoH with them. They discussed that the deeper understanding they acquired of the challenges faced by patients helped them to better meet patients at the point where they were in their journeys. This type of experience can also strengthen partnerships between programs and the community organizations to work together to help patients affected by SDoH.

This is a unique program for EM residents. A recent review found only 12 studies on SDoH in GME curricula—many including community site visits—but none included EM reidency programs.^2^ A program like this would likely also be beneficial to other residency programs and could be adjusted to include different relevant facilities. However, care would need to be taken not to dilute the intimate small-group nature of the tours that was so highly praised by residents.

## LIMITATIONS

One of the most significant limitations to this study is the small dataset and limited sample size. We implemented this intervention in one EM program in the Midwest with only 22 residents. We currently have two years of data, and we plan to have future classes participate in the program to strengthen the quality of data collected. Additionally, there had been data regarding residents’ confidence in providing community resources before the intervention. Finally, we believe that the true benefits of this program can best be appreciated in the qualitative data, which can be more challenging to analyze objectively than quantitative data. Future studies could evaluate the knowledge gained from this experience months after the retreat to better understand whether residents are incorporating their new-found knowledge into their clinical practice.

## CONCLUSION

Integrating social emergency medicine into daily practice is now recognized as a crucial component of providing high-quality care to our patients, but it is often overlooked in graduate medical education. In our study, residents reported that the dedicated time they spent to learn about and engage with their community promoted a deeper understanding of local resources and increased their confidence in integrating social determinants of health into their care of patients in the ED. The program is still in its early years; however, based on the positive feedback thus far, it continues as an annual event for our first-year EM residents. We believe it could likely be adapted to most EM residency programs.

## Figures and Tables

**Figure:**
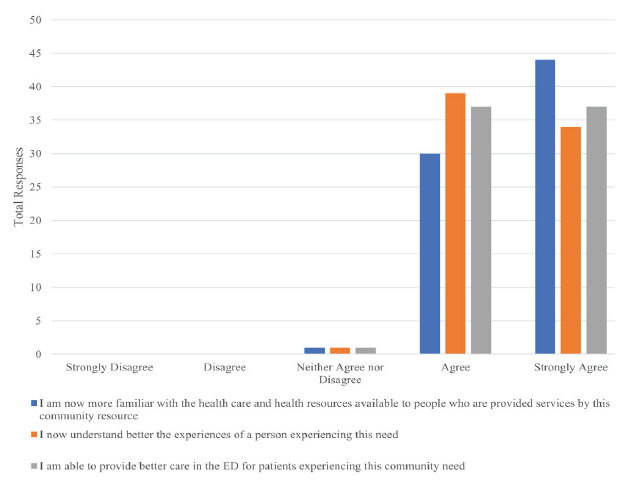
Combined feedback from first-year emergency medicine residents on five separate community site visits they made on Community Engagement Day 2021 and 2023. *ED*, emergency department.

**Table:** Organizations visited by first-year emergency medicine residents during their community engagement day.

1.	Treatment center focusing on substance use disorder
2.	Shelter for those experiencing homelessness and/or domestic violence
3.	County program for mental health
4.	Shelter for those experiencing homelessness
5.	Mental health emergency department
6.	Organization for food insecurity
7.	Organization providing resources to those who are unhoused
8.	Organization providing services to veterans with mental health disorders
